# 25-year trajectories of physical activity and atrial fibrillation risk: results from the ARIC study

**DOI:** 10.3389/fcvm.2024.1495504

**Published:** 2024-11-25

**Authors:** Tongxin Wang, Xinyi Wang, Tao Zhang, Jie Zhang, Runmin Lai, Jiqian Zhang, Dan Ma, Yan Jia, Qiyu Liu, Qiuyi Li, Jundi Jia, Wende Tian, Jianqing Ju, Hao Xu

**Affiliations:** ^1^ National Clinical Research Center for Chinese Medicine Cardiology, China Academy of Chinese Medical Sciences, Xiyuan Hospital, Haidian, Beijing, China; ^2^ Hong Kong Baptist University School of Chinese Medicine, Hong Kong, China; ^3^ National Integrated Traditional and Western Medicine Center for Cardiovascular Disease, China-Japan Friendship Hospital, Beijing, China; ^4^ Graduate School, Beijing University of Chinese Medicine, Beijing, China

**Keywords:** atherosclerosis risk in communities study, atrial fibrillation, physical activity, latent class trajectory modeling, risk factor

## Abstract

**Background:**

The relationship between serial changes in physical activity and the risk of developing atrial fibrillation (AF) has been rarely studied.

**Objectives:**

To evaluate the association between changes in physical activity over time and the incidence of AF.

**Methods:**

A total of 11,828 participants without AF at baseline (visit 1: 1987–1989) from the ARIC Study were included. Physical activity was measured using the modified Baecke Physical Activity Questionnaire at three different visits between 1987 and 2013. Physical activity trajectories over 25 years were identified using latent class trajectory modeling. The primary outcome was the incidence of AF. Multivariable Cox hazard regression models were used to evaluate the relationship between physical activity trajectories and the incidence of AF.

**Results:**

Over a median follow-up of 24 years, 2,108 AF cases (17.8%) occurred. Four distinct physical activity trajectories were identified: light [*n* = 5,266 (43.3%)]; reduced moderate [*n* = 3,583 (29.0%)]; moderate [*n* = 2,691 (25.0%)]; and vigorous intensity [*n* = 288 (2.8%)]. Compared to the light group, the hazard ratio (HR) and 95% confidence interval (CI) for AF were 1.18 (1.07–1.30) (*p* < 0.001) for the reduced moderate group, 0.61 (0.53–0.70) (*p* < 0.001) for the moderate group, and 0.82 (0.59–1.12) (*p* = 0.21) for the vigorous group, after multivariate adjustments.

**Conclusion:**

Maintaining moderate levels of physical activity is associated with a lower risk of AF, while a decrease in activity from moderate to light levels increases the risk. These findings highlight the importance of sustaining adequate physical activity levels for the prevention of AF.

## 1 Introduction

Atrial fibrillation (AF) is a common cardiac arrhythmia with significant implications for public health and healthcare systems, particularly in older adults (1, 2). The incidence of AF is strongly linked to the inadequate management of risk factors in specific populations (3–5). Physical activity is a crucial component of lifestyle interventions, with moderate levels of activity associated with reduced mortality in the elderly (6). Current guidelines emphasize the positive relationship between regular physical activity and cardiovascular health (7). However, research on the association between physical activity and AF risk has produced mixed results. Some studies have observed U-shaped or J-shaped patterns, suggesting that both low and very high levels of physical activity elevate the risk of AF, while moderate activity provides protective effects (8–10). Conversely, other studies propose a more linear relationship, where increasing levels of physical activity consistently decrease AF risk (11). These varying findings highlight the complexity of the relationship between physical activity and AF, highlighting the need for further research on how long-term physical activity patterns influence AF risk. Most notably, prior studies have predominantly relied on single-point assessments of physical activity, often failing to account for longitudinal changes over time (8–11). Therefore, our study aims to address this gap by investigating the impact of long-term physical activity patterns on AF risk using data from the Atherosclerosis Risk in Communities (ARIC) Study.

## 2 Method

### 2.1 Study population

The ARIC study is a population-based prospective cohort study designed to investigate the causes and risk factors associated with cardiovascular disease. The study initially recruited 15,792 participants, aged 45–64 years, from four distinct U.S. communities between 1987 and 1989. Follow-up visits occurred in 1990–1992 (visit 2), 1993–1995 (visit 3), 1996–1998 (visit 4), 2011–2013 (visit 5), 2016–2017 (visit 6), and 2018–2019 (visit 7). The study received approval from the ethical committees at each participating site, and all participants provided written informed consent.

Participants with prevalent AF or missing AF data at baseline (*n* = 243), missing baseline physical activity data (*n* = 26), only one record of physical activity (*n* = 2,435), or missing covariate data (*n* = 1,186) were excluded. Additionally, participants identified as Asian (*n* = 46) and African Americans from Washington County, MD (*n* = 32) or Minneapolis, MN (*n* = 22) were excluded, as in previous studies (12, 13). This left a total of 11,828 participants eligible for analysis (Supplementary Figure S1).

### 2.2 Assessment of physical activity

Physical activity was assessed at visits 1, 3, and 5 using the interviewer-administered modified Baecke Physical Activity Questionnaire. Participants reported their leisure-time physical activities, including the frequency and duration of each activity. The questionnaire's content and format remained consistent across all three visits, and previous studies have validated its reliability and accuracy (14, 15). Each activity was assigned a metabolic equivalent (MET) score to reflect its intensity (7, 16). MET scores ranged from 1 to 12, with higher values indicating more intense activities. For instance, a MET value of 3 corresponds to walking at 3 miles per hour, while a value of 8.8 corresponds to jogging at 5.6 miles per hour. Activities were categorized into light (<3 METs), moderate (3–5.9 METs), or vigorous (≥6 METs) intensity levels. A composite category of moderate to vigorous intensity included any activity with a MET score of 3 or higher (7).

To quantify total physical activity, we created a variable representing MET-minutes per week (MET·min·wk^−1^) for continuous analysis. Physical activity trajectory classes were estimated using summary scores derived from visit 1 (baseline), visit 3 (approximately 6 years later), and visit 5 (approximately 25 years later). To classify activity trajectories, we followed the 2018 Physical Activity Guidelines for Americans (17) and the 2011 Guidance for Prescribing Exercise (18). Based on these guidelines, we categorized physical activity intensity into three levels: low (0 to <600 MET·min·wk^−1^), moderate (600 to <1,200 MET·min·wk^−1^), and high (≥1,200 MET·min·wk^−1^).

### 2.3 AF identification

Atrial fibrillation (AF) was identified through electrocardiograms (ECGs) performed during study visits, hospital discharge codes, and death certificates, as previously described (19). All ECGs automatically coded as AF or atrial flutter were visually confirmed by a cardiologist. Trained abstractors reviewed participants’ hospitalization records and collected information on all International Classification of Diseases, Ninth Revision, Clinical Modification (ICD-9-CM) codes for diagnoses. AF was considered present if the ICD-9-CM code 427.31 (AF) or 427.32 (atrial flutter) was listed in any hospitalization record, except for those associated with open cardiac surgery. Additionally, AF was defined if the ICD-9-CM code 427.31 or 427.32 was listed as a cause of death.

### 2.4 Assessment of covariates

All covariates were assessed during visit 1. Participants self-reported their race, age, sex, education level, smoking status, and alcohol consumption. Height and weight were measured with participants wearing light clothing, and body mass index (BMI) was calculated as weight (in kilograms) divided by height squared (in meters). Seated blood pressure was measured using the average of the last two of three readings, taken with a random-zero sphygmomanometer after a 5-minute rest period. Hypertension was defined as a systolic blood pressure (SBP) of at least 140 mm Hg and/or a diastolic blood pressure (DBP) of at least 90 mm Hg, or the use of antihypertensive medication within the past two weeks (13). Diabetes (DM) was defined as a fasting blood glucose level of at least 126 mg/dl, a non-fasting blood glucose level of at least 200 mg/dl, the use of antidiabetic medication, or a self-reported physician diagnosis (13). Stroke was identified based on the presence of six symptoms: speech difficulties, vision problems, double vision, numbness, paralysis, and dizziness, corresponding to the artery affected (17). Prevalent coronary heart disease (CHD) and heart failure (HF) were determined using established criteria (20, 21).

### 2.5 Statistical analysis

Physical activity trajectories from 1987 to 2012 were identified using latent mixed modeling with the Stata traj program (22). Latent class trajectory modeling is a variant of finite mixture modeling that uses maximum likelihood estimation to identify latent groups of individuals who follow similar patterns of a variable over time (23). Trajectory shapes were determined by progressively reducing the polynomial function's degree, starting with a cubic function, until each growth parameter estimate was statistically significant. The optimal number of trajectories was selected based on the minimum Bayesian Information Criterion (BIC) (24) and a class size of at least 2% of the population, while ensuring a close fit between estimated and actual trajectories. The Average Posterior Probability (AvePP) for each group was required to exceed 0.7. Second-order polynomials were employed in our models. Participants were assigned to the trajectory group with the highest posterior predictive probability (Supplementary Table S1).

Continuous variables were presented as mean [standard deviation (SD)], and categorical variables as numbers and percentages. Baseline characteristics across physical activity trajectory classes were compared using analysis of variance (ANOVA) F-tests for continuous variables and Chi-square tests for categorical variables.

The Kaplan-Meier method was used to calculate the cumulative incidence of AF across physical activity trajectories, with the log-rank test employed to assess differences. Multivariable Cox proportional hazards regression was used to estimate hazard ratios (HRs) and 95% CIs for the association between physical activity trajectories and incident AF. Model 1 adjusted for age, sex, and race. Model 2 included additional adjustments for BMI, height, weight, SBP, and DBP at baseline. Model 3 was further adjusted for education, smoking status, alcohol consumption, and history of hypertension, stroke, DM, CHD, and HF at baseline.

Sensitivity analyses were performed to test the robustness of the trajectory classifications and their association with AF risk. These included additional adjustments and exclusions as follows: (a) exclusion of patients with baseline hypertension, stroke, DM, CHD, or HF; (b) exclusion of patients with baseline hypertension, stroke, DM, CHD, HF, or current/former smoking status; and (c) exclusion of patients with baseline hypertension, stroke, DM, CHD, HF, or current/former alcohol consumption (8).

All analyses were conducted using Stata version 17 and R version 4.2.3. Statistical significance was defined as *p* < 0.05 (two-sided test).

## 3 Results

### 3.1 Baseline characteristics

The study included 11,828 participants, comprising 9,205 white and 2,623 black individuals, with a mean age of 54.0 ± 5.7 years at baseline. Baseline characteristics of the study population are shown in Table 1. Over a median follow-up period of 24 years (interquartile range, 18.8–25.5 years), 2,108 participants (1,126 men and 982 women) were diagnosed with AF. Participants in the light physical activity group had a higher average BMI (28.6 kg/m^2^) compared to those in the vigorous intensity group (25.3 kg/m^2^). Other baseline factors, including blood pressure, smoking status, and the prevalence of conditions such as hypertension and coronary heart disease, varied across the physical activity trajectory groups.

**Table 1:** Baseline characteristics of the study population.

	Light	Reduced Moderate	Moderate	Vigorous intensity
N	5,266	3,583	2,691	288
Age (years)	54.02 (5.657)	55.86 (5.643)	51.8 (5.031)	51.8 (5.129)
Female	3,195 (60.67%)	1,779 (49.65%)	1,435 (53.33%)	101 (35.07)
African American	1,681 (31.92%)	521 (14.54%)	396 (14.72%)	25 (8.68%)
Body height (cm)	167.5 (9.026)	169.1 (9.334)	169.4 (9.558)	173.4 (8.898)
Body weight (LB)	176.8 (38.83)	171.9 (35.12)	166.5 (33.22)	167.9 (30.42)
BMI (Kg/m^2^)	28.62 (5.818)	27.24 (4.764)	26.26 (4.146)	25.28 (3.515)
SBP (mmHg)	122.0 (18.64)	121.2 (17.41)	114.7 (15.18)	115.4 (14.46)
DBP (mmHg)	74.16 (11.35)	72.87 (10.42)	71.87 (9.957)	72.43 (9.859)
Education				
Basic or 0 year	1,539 (29.23%)	597 (16.66%)	217 (8.06%)	5 (1.74%)
Intermediate	2,313 (43.92%)	1,506 (42.03%)	1,059 (39.35%)	70 (24.31%)
Advanced	1,414 (26.85%)	1,480 (40.31%)	1,415 (52.58%)	213 (73.96%)
Smoking				
Current smoker	1,518 (28.83%)	763 (21.30%)	433 (16.09%)	19 (6.60%)
Former smoker	1,492 (28.33%)	1,401 (39.10%)	951 (35.34%)	132 (45.83%)
Never smoker	2,256 (42.84%)	1,419 (39.60%)	1,307 (48.57%)	137 (47.57%)
Drinking				
Current drinker	2,622 (49.79%)	2,253 (62.88%)	1,821 (67.67%)	227 (78.82%)
Former drinker	1,061 (20.15%)	593 (16.55%)	354 (13.16%)	38 (13.19%)
Never drinker	1,583 (30.06%)	737 (20.57%)	516 (19.18%)	23 (7.99%)
CHD	200 (3.80%)	221 (6.17%)	41 (1.52%)	10 (3.47%)
Hypertension	2,043 (38.80%)	1,193 (33.30%)	507 (18.84%)	51 (17.71%)
Stroke	90 (1.71%)	41 (1.14%)	12 (0.45%)	1 (0.35%)
DM	691 (13.12%)	416 (11.61%)	100 (3.72%)	5 (1.74%)
HF	282 (5.36%)	135 (3.77%)	42 (1.56%)	4 (1.39%)

BMI, body mass index; SBP, systolic blood pressure; DBP, diastolic blood pressure; CHD, coronary heart disease; DM, diabetes; HF, heart failure.

### 3.2 Association between physical activity trajectories and incident AF

**Figure 1:**
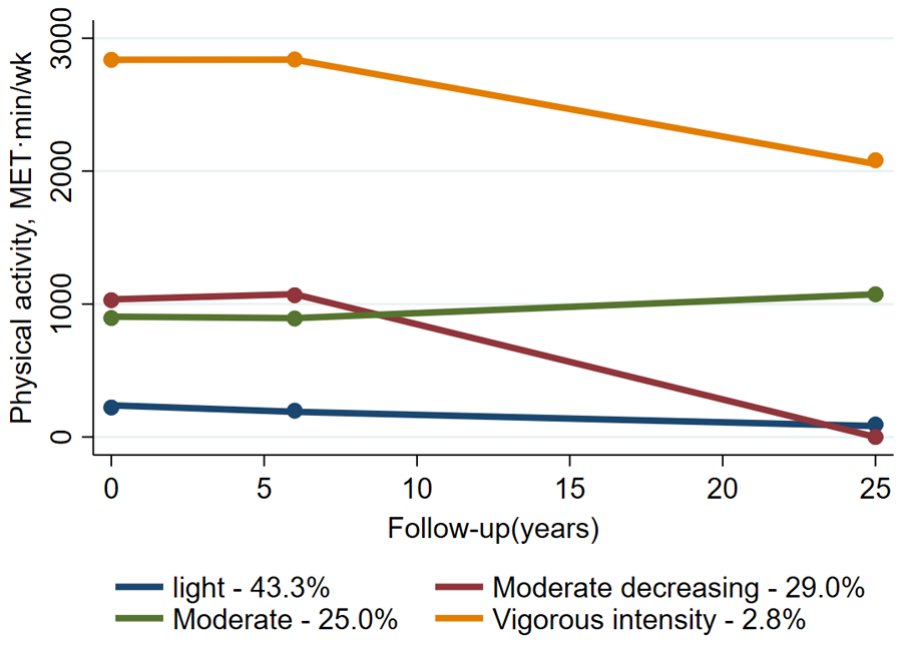
Trajectories of physical activity from visit 1-visit 5.

This study identified four distinct physical activity trajectories: (1) Maintaining a light physical activity level throughout the follow-up period [referred to as “light”; *n* = 5,266 (43.3%)], (2) Starting with a moderate level of physical activity but progressively reducing it [referred to as “reduced moderate”; *n* = 3,583 (29.0%)], (3) Maintaining a moderate physical activity level throughout the follow-up period [referred to as “moderate”; *n* = 2,691 (25.0%)], and (4) Maintaining a vigorous intensity physical activity level throughout the follow-up period [referred to as “vigorous intensity”; *n* = 288 (2.8%)] (Figure 1).

**Figure 2:**
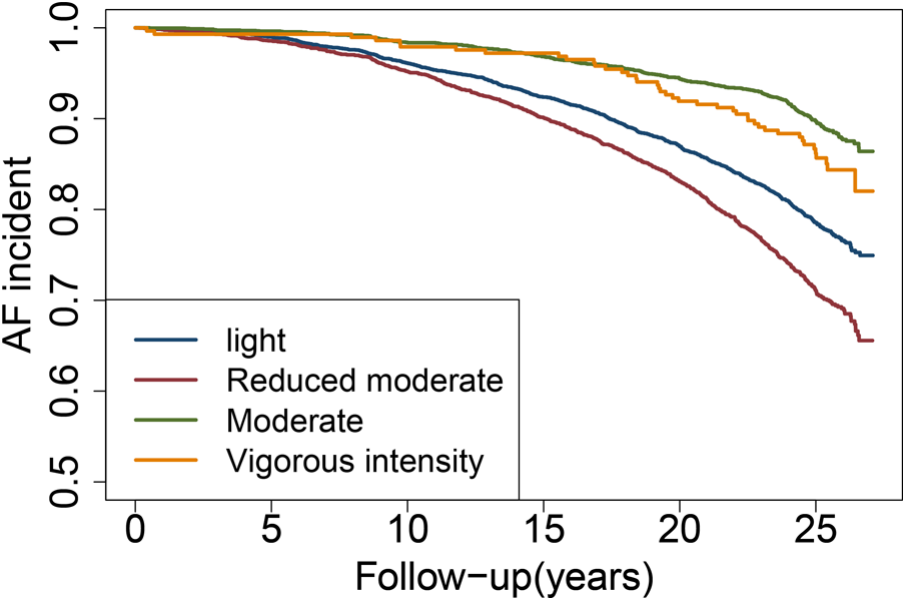
Cumulative incidence of incident AF by physical activity trajectories.

The incidence rates of AF were 18.00%, 23.05%, 10.85%, and 14.58% for the light, reduced moderate, moderate, and vigorous intensity groups, respectively (Supplementary Figure S2). Kaplan-Meier curve analysis indicated that, compared to the light group, the reduced moderate group had an increased risk of developing AF, while the moderate and vigorous intensity groups showed a decreased risk (log-rank *p* < 0.001) (Figure 2).

Multivariate Cox regression analysis revealed that, compared to the light group, participants in the moderate group had a 39% lower risk of AF (HR 0.61, 95% CI 0.53–0.70; *p* < 0.001), whereas those in the reduced moderate group had an 18% higher risk (HR 1.18, 95% CI 1.07–1.30; *p* < 0.001) (Table 2). After full adjustment, the vigorous intensity group exhibited a reduced risk of incident AF compared to the light group, but an increased risk compared to the moderate intensity group, though these associations were not statistically significant (*p* > 0.05) (Table 2, Supplementary Table S2).

**Table 2:** Trajectories of physical activity and risk of atrial fibrillation.

	Case/N (%)	Risk of AF					
		Model 1	*P*-value	Model 2	*P*-value	Model 3	*P*-value
Light	948/5,266 (18)	1.00 (reference)		1.00 (reference)		1.00 (reference)	
Reduced Moderate	826/3,583 (23)	1.08 (0.98–1.19)	0.13	1.13 (1.03–1.25)	0.01	1.18 (1.07–1.30)	<0.001
Moderate	292/2,691 (11)	0.47 (0.41–0.54)	<0.001	0.53 (0.46–0.61)	<0.001	0.61 (0.53–0.70)	<0.001
Vigorous intensity	42/288 (15)	0.59 (0.43–0.81)	<0.001	0.67 (0.49–0.91)	0.01	0.82 (0.59–1.12)	0.21

Data are hazard ratios (95% CIs). Cases/N = number of AF cases/number of total individuals at risk per trajectory.

Model 1: age, sex and race at baseline.

Model 2: Model 1 + BMI, height, weight, SBP, DBP at baseline.

Model 3: Model 2 + education + smoking + drinking + hypertension + stroke + DM + CHD + HF at baseline.

Stratification by age (≤54 or >54 years), sex (male or female), race (white or black), BMI (<30 or ≥30 kg/m^2^), smoking status (current, former, or never), and drinking status (current, former, or never) showed that the relationship between physical activity trajectories and incident AF remained generally consistent (Supplementary Figure S3).

Sensitivity analyses, which excluded participants with CVD or current/former smoking or drinking habits, did not significantly change the observed associations (Supplementary Figure S4, Table S3).

## 4 Discussion

This study, which analyzed 11,828 middle-aged to elderly individuals, identified four distinct physical activity trajectories: light, reduced moderate, moderate, and vigorous intensity. The findings revealed that individuals following a reduced moderate physical activity trajectory had an increased risk of developing AF compared to those maintaining a long-term light physical activity trajectory. Conversely, participants on a moderate physical activity trajectory exhibited a lower likelihood of incident AF. While the association for the vigorous activity trajectory showed a trend toward reduced AF risk, it was not statistically significant.

The observation that the moderate physical activity trajectory was associated with a lower risk of incident AF aligns with previous studies, which have demonstrated a U-shaped or J-shaped relationship between physical activity and AF. These studies suggest that both low and high levels of physical activity are linked to an increased risk of AF (8, 11, 25–27). In the context of a U-shaped relationship, moderate physical activity levels provide protective benefits, whereas both excessively low and high levels may increase cardiovascular stress and predispose individuals to AF. Specifically, higher levels of endurance exercise have been associated with structural changes in the atria, such as atrial dilation and fibrosis, which may increase the likelihood of developing AF (28, 29).

Moderate-intensity physical activity not only lowers the risk of AF but also reduces the incidence of other cardiovascular diseases, including hypertension (30), metabolic syndrome (31), CHD (32), stroke (7), and cardiovascular mortality (33). The reduction in disease risk is likely due to the beneficial effects of exercise on various physiological factors, including blood pressure regulation, lipid profile improvement, glucose metabolism, and reductions in inflammatory markers (34). Overall, moderate-intensity aerobic exercise is a safe and effective method for improving cardiovascular health and reducing the risk of multiple chronic diseases. Clinicians should encourage patients to engage in regular physical activity as part of a comprehensive strategy for the prevention and management of cardiovascular disease.

Moderate-intensity physical activity can be achieved through various aerobic exercises, including brisk walking, cycling, swimming, dancing, and hiking. The American College of Sports Medicine recommends that adults engage in at least 150 min of moderate-intensity aerobic exercise per week, distributed over a minimum of three days (18). This can be accomplished through structured exercise sessions or by incorporating physical activity into daily routines, such as taking the stairs instead of the elevator or walking to work (18).

The group with reduced moderate physical activity showed a higher risk of incident AF compared to the light activity group. Bauman et al. (35) found that reducing physical activity from moderate to low levels was associated with an increased risk of CVD deaths compared to maintaining low levels of physical activity in the elderly. This suggests that lowering moderate activity levels, even if previously sufficient, may raise the risk of cardiovascular events if not maintained at an adequate level. The higher risk of AF in individuals following the reduced moderate activity trajectory could be attributed to insufficient physical activity to provide a protective effect, while still being exposed to potential harms, such as increased inflammation and oxidative stress associated with physical inactivity (33, 36). Additionally, evidence suggests that the cardioprotective benefits of physical activity may not be sustained without consistent exercise. When physical activity is reduced or stopped, these protective effects may diminish quickly (37, 38). However, the exact mechanisms behind this have yet to be fully explored.

There is ongoing debate about the impact of PA on AF. While the prevailing view holds that PA supports cardiovascular health and lowers AF risk, some studies have found no statistically significant association between regular PA and increased AF incidence, including two meta-analyses. Kunutsor et al. conducted a meta-analysis involving approximately 2 million participants and found no significant relationship between PA and AF risk in the general population, though they observed sex-specific differences (39). Similarly, Ofman et al. reported no significant association between PA and AF, challenging the assumption that regular PA provides protection against AF (40). In contrast, our study employed latent class trajectory analysis, allowing for a more nuanced classification of participants based on long-term PA patterns. This method enabled us to identify more precise associations between PA intensity and AF risk. We found that maintaining moderate-intensity PA significantly reduces AF risk compared to light-intensity PA, and that reducing PA from moderate to light increases the risk. Importantly, consistently maintaining moderate-intensity PA mitigates this risk. These findings highlight the importance of sustained moderate-intensity PA in preventing AF and may help explain why previous studies failed to find significant associations between PA and AF risk.

Notably, this study did not identify any trajectories where participants increased their activity levels from low to moderate or high. This may be due to the tendency for older adults who engaged in light physical activity during middle age to experience fewer improvements in physical health later in life, consistent with findings from prior research (41). While studies on increasing PA among the elderly have shown mixed results, some have demonstrated potential cardiovascular benefits. Therefore, it is important to consider both the potential benefits and risks when encouraging increased PA in older adults, particularly those with pre-existing conditions. A balanced approach, with tailored recommendations, is advised to maximize benefits while minimizing risks.

Another finding of this study is that individuals maintaining a vigorous intensity level of physical activity may have a lower risk of incident AF compared to those maintaining a light level of activity. Vigorous intensity physical activity has shown both beneficial and adverse effects on cardiovascular health, particularly in middle-aged and elderly individuals. On one hand, vigorous activity is associated with improved cardiovascular fitness, reduced inflammation, and a lower risk of conditions such as CHD, stroke, and HF (36, 42, 43). On the other hand, vigorous intensity activity may increase the risk of AF, a common arrhythmia in the aging population (29, 44). Several studies have reported an increased risk of arrhythmias in individuals participating in high-endurance activities, such as marathon running and triathlons (45, 46). However, the relationship between vigorous intensity activity and AF remains inconsistent. Some studies suggest a positive association between vigorous activity and AF risk (8, 47), while others report no association or even a protective effect (48, 49). Further research is needed to determine the optimal amount and intensity of physical activity to reduce AF risk in the general population.

The subgroup analysis by age and gender revealed notable findings. Among individuals over 54 years of age, while the overall trends mirrored those of the general population, the association between reduced moderate and light physical activity was not statistically significant (Supplementary Materials). This observation aligns with studies suggesting that, with advancing age, factors such as decreased muscle mass, increased vascular stiffness, and reduced aerobic capacity may lessen the protective effects of physical activity on cardiovascular health (1, 50, 51). Regarding gender, both males and females showed trends consistent with the overall population. However, in males, the association between reduced moderate and light physical activity was not statistically significant, which is worth noting (Supplementary Materials). Prior research indicates that men may experience less pronounced benefits from changes in physical activity compared to women, potentially due to differences in hormonal responses and cardiovascular adaptations (52).

To our knowledge, this is the first study to explore the longitudinal patterns of change in physical activity and their potential correlation with AF incidence. These findings highlight the importance of maintaining a moderate level of physical activity throughout adulthood to support cardiovascular health and reduce the risk of developing AF. Our study has several notable strengths. First, it is based on a large, community-based cohort. Second, repeat visits were conducted with consistent assessments of risk factors over time, and long-term annual follow-up was carried out over 25 years. This design allowed us to capture health trends over an extended period.

However, there are several limitations to consider. First, the primary population in this study consisted of middle-aged to elderly individuals, which may limit the generalizability of the findings to younger populations. Additionally, the ARIC database did not capture records of palpitation symptoms, meaning some individuals may have reduced their physical activity due to these symptoms, complicating the interpretation of causality. As such, the causal relationship between the reduced moderate activity trajectory and incident AF remains uncertain. We conducted several sensitivity analyses and subgroup analyses to further investigate the findings, and the results remained largely consistent. However, as with any observational study, residual confounding cannot be completely ruled out, despite our careful adjustments for both well-known and suspected risk factors. Lastly, because this study focused on middle-aged to elderly participants, it is unclear whether these conclusions apply to younger populations.

## 5 Conclusions

Moderate physical activity was associated with a reduced risk of AF, whereas a decrease from moderate intensity led to a higher risk. Although the findings for vigorous physical activity were not statistically significant, the potential protective effects should not be overlooked. Our results suggest that the cardioprotective benefits of physical activity may diminish without consistent exercise. This study emphasizes the importance of maintaining regular moderate physical activity to prevent AF, particularly in middle-aged and elderly populations.

## Data Availability

The datasets presented in this article are not readily available due to strict agreement with the database providers that prohibits disclosing specific data details to third parties. Further inquiries can be directed to the corresponding authors.
