# Supplementary material for: 25-year trajectories of physical activity and atrial fibrillation risk: results from the ARIC study
Source: Front Cardiovasc Med. 2024 Nov 25;11:1495504. doi: 10.3389/fcvm.2024.1495504 (PMC11626529; doi:10.3389/fcvm.2024.1495504)
Supplement: Supplementary file 1 [file Datasheet1.docx]

**Supplementary material**

Figure S1. Flow chart of selection of ARIC study participants.

Table S1. Group-based trajectory model fit summary.

Figure S2. Prevalence of incident AF across the physical activity trajectory groups.

Table S2: Trajectories of physical activity and risk of AF (Moderate group as control group)

Figure S3. Subgroup analysis of the association between physical activity trajectories and incident AF.

Figure S4. Sensitivity analyses: Trajectories of physical activity from visit1-visit5.

Table S3. Sensitivity analyses: Risk of AF in relation to physical activity trajectories in various models.


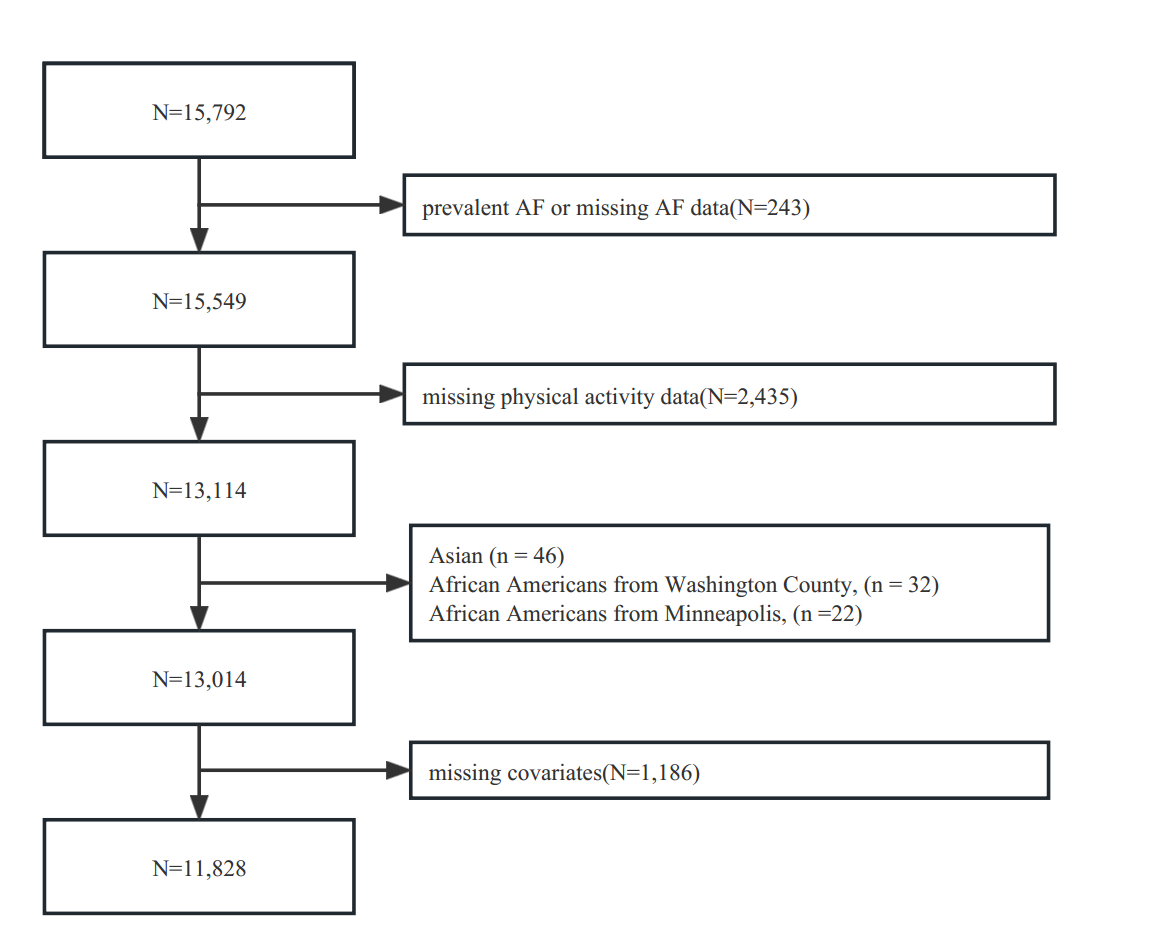


**Figure S1.** Flow chart of selection of ARIC study participants

**Table S1.** Group-based trajectory model fit summary (N=11828)

| Number of groups | BIC | AIC | group % | AvePP |
| --- | --- | --- | --- | --- |
| 2 | -171765.53 | -171736.01 | 80.88% | 0.93 |
|  |  |  | 19.12% | 0.82 |
| 3 | -171359.11 | -171314.84 | 61.92% | 0.86 |
|  |  |  | 19.84% | 0.72 |
|  |  |  | 18.24% | 0.87 |
| 4 | -170859.65 | -170796.07 | 43.26% | 0.82 |
|  |  |  | 28.96% | 0.77 |
|  |  |  | 24.98% | 0.88 |
|  |  |  | 2.81% | 0.88 |
| 5 | -170903.98 | -170830.20 | 17.20% | 0.72 |
|  |  |  | 44.89% | 0.82 |
|  |  |  | 29.72% | 0.94 |
|  |  |  | 4.78% | 0.78 |
|  |  |  | 3.41% | 0.88 |

*AIC* Akaike’s information criterion, *BIC* Bayesian information criterion, *AvePP* Average posterior probability


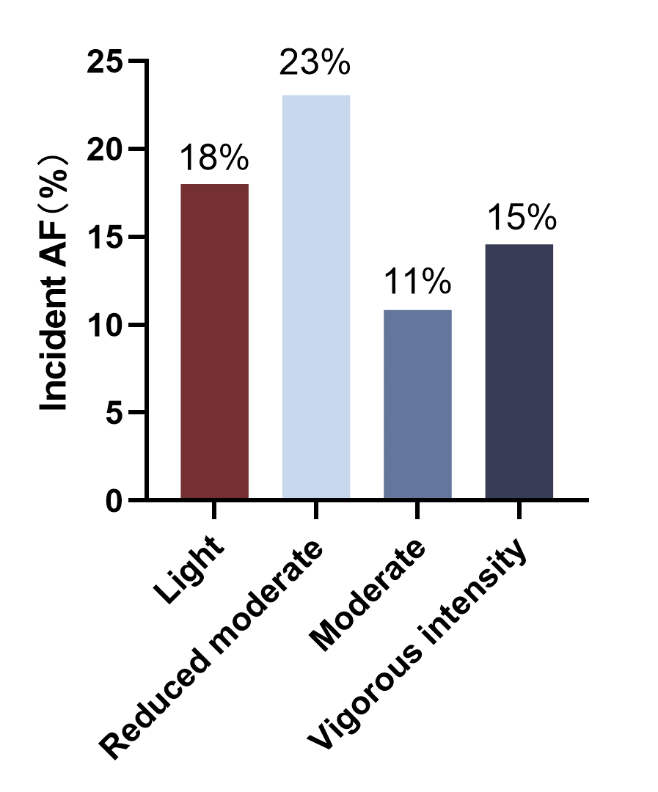


**Figure S2.** Prevalence of incident AF across the physical activity trajectory groups

**Table S2:** Trajectories of physical activity and risk of AF (Moderate group as control group)

|  | Case/N (%) | Risk of AF | | | | | |
| --- | --- | --- | --- | --- | --- | --- | --- |
|  |  | Model 1 | *p* value | Model 2 | p value | Model 3 | *p* value |
| light | 948/5266(18) | 2.12(1.86-2.43) | <0.001 | 1.88(1.64-2.16) | <0.0001 | 1.64(1.43-1.89) | <0.001 |
| Reduced Moderate | 826/3583(23） | 2.29(1.99-2.62) | <0.001 | 2.13(1.86-2.45) | <0.0001 | 1.94(1.69-2.23) | <0.001 |
| Moderate | 292/2691(11) | 1.00(reference) |  | 1.00(reference) | <0.0001 | 1.00(reference) |  |
| Vigorous intensity | 42/288(15) | 1.25(0.91-1.73) | 0.17 | 1.26(1.07-1.09 | 0.16 | 1.34(0.97-1.86) | 0.08 |

Data are hazard ratios (95% CIs). Cases/N=number of AF cases/number of total individuals at risk per trajectory.

Model 1 was adjusted for age, sex and race.

Model 2: Model 1 + BMI, height, weight, SBP, DBP at baseline.

Model 3: Model 2 + education, smoking, drinking, hypertension, stroke, DM, CHD, HF at baseline.


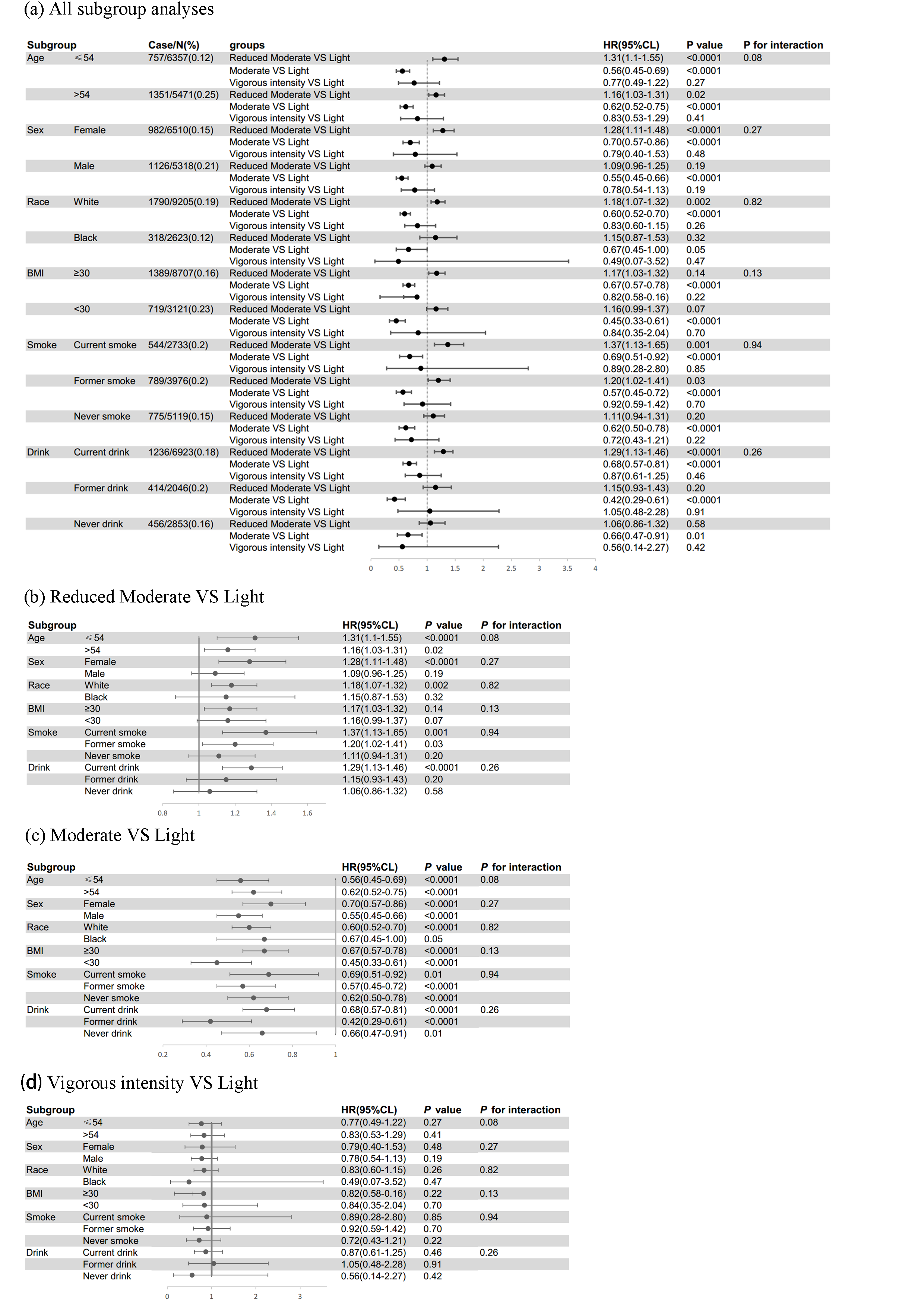


**Figure S3 a.** Subgroup analysis of the association between physical activity trajectories and incident AF

Cox regression, adjusted for baseline body mass index (BMI), height, weight, systolic blood pressure (SBP), diastolic blood pressure (DBP), education, smoking status, drinking status, hypertension, stroke, diabetes mellitus (DM), coronary heart disease (CHD), and heart failure (HF), was performed on subgroups according to age (≤ 54 or > 54 years), sex (male or female), race (White or Black), BMI (< 30 or ≥ 30 kg/m2), smoking status (current, former, or never), and drinking status (current, former, or never).

(a) Excluding baseline CVD.


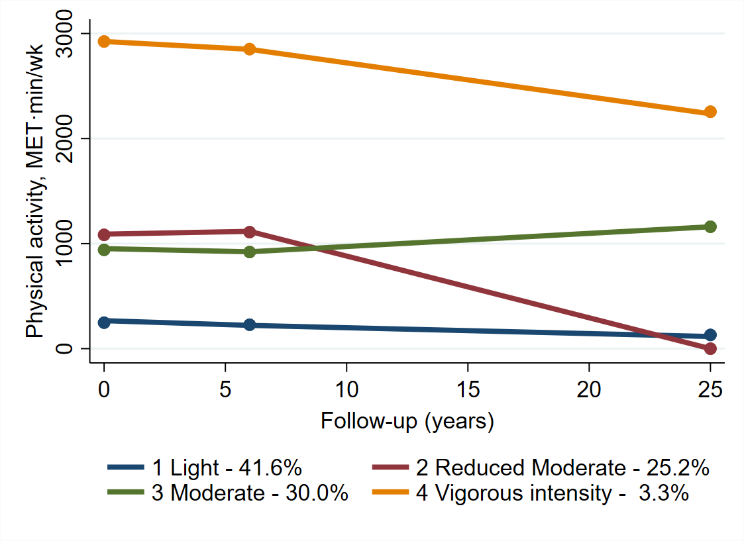


(b) Excluding baseline CVD + current or former smokers.


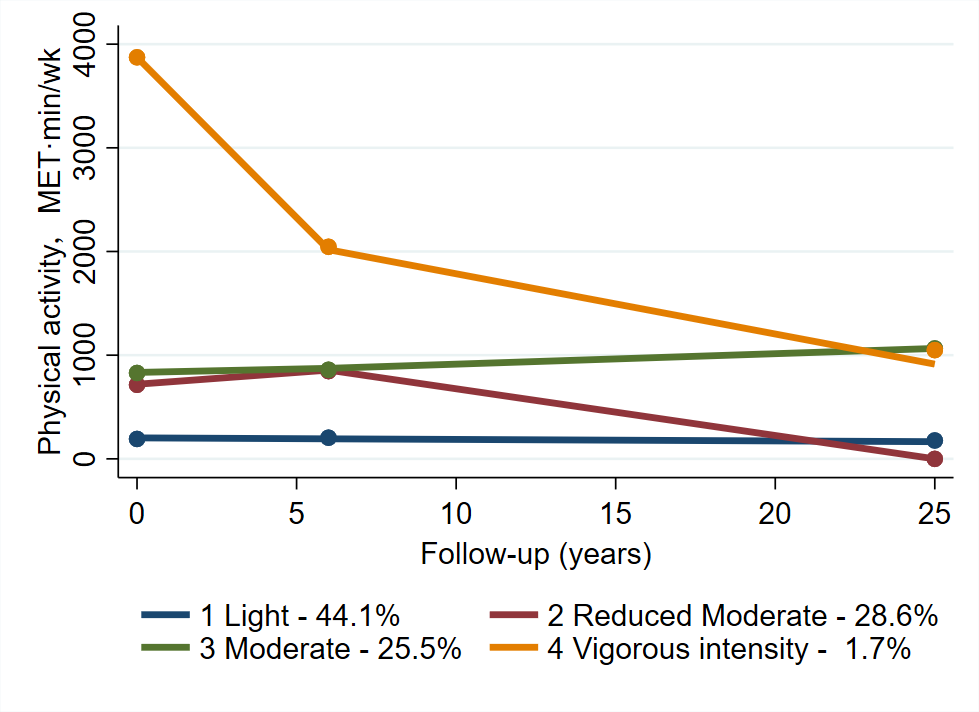


(c) Excluding baseline CVD + current or former drinkers.


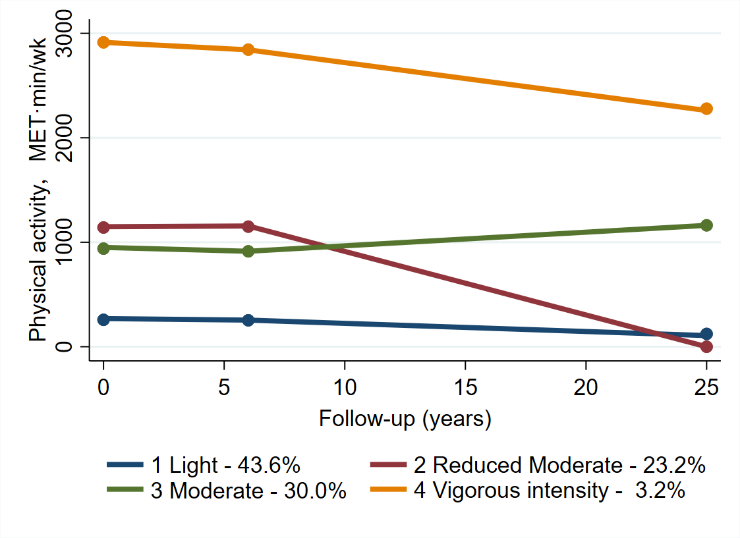


**Figure** **S4**. Sensitivity analyses: Trajectories of physical activity from visit1-visit5

**Table S3**. Sensitivity analyses: Risk of AF in relation to physical activity trajectories in various models

(a) Excluding baseline CVD.

| Case/N (%) | Risk of AF | | | | | |
| --- | --- | --- | --- | --- | --- | --- |
| 1015/7,234(14%) | Model 1a | *p* value | Model 2a | *p* value | Model 3a | *p* value |
| light | 1.00(reference) |  | 1.00(reference) |  | 1.00(reference) |  |
| Moderate decreasing | 1.13(0.99-1.31) | 0.07 | 1.15(1.00-1.33) | 0.05 | 1.20(1.04-1.39) | 0.02 |
| Moderate | 0.58(0.49-0.69) | <0.001 | 0.61(0.51-0.72) | <0.001 | 0.66(0.55-0.78) | <0.001 |
| Vigorous intensity | 0.75(0.43-0.81) | 0.24 | 0.80(0.55-1.17) | 0.24 | 0.93(0.63-1.36) | 0.71 |

Model 1a: was adjusted for age, sex and race.

Model 2a: Model 1 + BMI, height, weight, SBP, DBP at baseline.

Model 3a: Model 2 + education, smoking, drinking at baseline.

(b) Excluding baseline CVD + current or former smokers.

| Case/N (%) | Risk of AF | | | | | |
| --- | --- | --- | --- | --- | --- | --- |
| 379/3,119 (12%) | Model 1b | *p* value | Model 2b | *p* value | Model 3b | *p* value |
| light | 1.00(reference) |  | 1.00(reference) |  | 1.00(reference) |  |
| Moderate decreasing | 1.16(0.92-1.47) | 0.21 | 1.20(0.94-1.53) | 0.13 | 1.23(0.96-1.56) | 0.10 |
| Moderate | 0.67(0.51-0.88) | 0.004 | 0.71(0.54-0.94) | 0.02 | 0.73(0.55-0.96) | 0.03 |
| Vigorous intensity | 0.81(0.45-1.47) | 0.51 | 0.81(0.45-1.49) | 0.51 | 0.82(0.45-1.51) | 0.53 |

Model 1b: was adjusted for age, sex and race.

Model 2b: Model 1 + BMI, height, weight, SBP, DBP at baseline.

Model 3b: Model 2 + education, drinking at baseline.

(c) Excluding baseline CVD + current or former drinkers.

| Case/N (%) | Risk of AF | | | | | |
| --- | --- | --- | --- | --- | --- | --- |
| 187/1537(12%) | Model 1c | *p* value | Model 2c | *p* value | Model 3c | *p* value |
| light | 1.00(reference) |  | 1.00(reference) |  | 1.00(reference) |  |
| Moderate decreasing | 1.07(0.93-1.24) | 0.32 | 1.08(0.93-1.25) | 0.31 | 1.13(0.97-1.30) | 0.11 |
| Moderate | 0.56(0.47-0.67) | <0.001 | 0.59(0.49-0.70) | <0.001 | 0.64(0.53-0.76) | <0.001 |
| Vigorous intensity | 0.77(0.53-1.12) | 0.18 | 0.77(1.09-1.12) | 0.18 | 0.90(0.61-1.31) | 0.58 |

Model 1c: was adjusted for age, sex and race.

Model 2c: Model 1 + BMI, height, weight, SBP, DBP at baseline.

Model 3c: Model 2 + education, smoking at baseline.
